# Characterization of Stanniocalcin-1 Receptors in the Rainbow Trout

**DOI:** 10.5402/2012/257841

**Published:** 2012-02-06

**Authors:** Timothy D. J. Richards, Amanda L. Fenton, Rahma Syed, Graham F. Wagner

**Affiliations:** ^1^Department of Physiology and Pharmacology, Faculty of Medicine and Dentistry, University of Western Ontario, London, ON, Canada N6A 5C1; ^2^Department of Biology, Faculty of Science, University of Western Ontario, London, ON, Canada N6A 5C1

## Abstract

Mammalian stanniocalcin-1 (STC-1) is one of several ligands targeted to mitochondria. High affinity STC-1 receptors are present on the mitochondrial membranes of nephron cells, myocytes, and hepatocytes, to enable ligand sequestration within the matrix. However, STC-1 receptors have not been characterized in fish. Nor is it known if mitochondrial targeting occurs in fish. The aim of the study was to address these questions. Saturation binding assays were carried out to obtain estimates of *K*_D_ and *B*_max_. They revealed the presence of saturable, high-affinity receptors on both membranes and mitochondria of liver, muscle, and gill filament. In situ ligand binding (ISLB) was used to localize receptors at the histological level and revealed some unexpected findings. In cranium, for instance, receptors were found mainly in the cartilage matrix, as opposed to the chondrocytes. In brain, the majority of receptors were located on neuropil areas as opposed to neuronal cell bodies. In skeletal muscle, receptors were confined to periodic striations, tentatively identified as the Z lines. Receptors were even found on STC-1 producing corpuscles of Stannius cells, raising the possibility of there being an autocrine feedback loop or, perhaps, a soluble binding protein that is released with the ligand to regulate its bioavailability.

## 1. Introduction

Although stannniocalcin-1 (STC-1) was originally described in fish, it is now known to be present throughout the animal kingdom in both vertebrates and invertebrates [1]. The principle source of STC-1 in bony fish are endocrine glands known as the corpuscles of Stannius (CS) which are anatomically associated with the kidneys. STC-1 release is stimulated by a rise in serum levels of ionic calcium above the physiological set point through the activation of calcium-sensing receptors. The hormone then exerts regulatory effects on the epithelial transport of calcium and/or phosphate across the gills, gut, and kidneys in order to restore normocalcemia [2].

There is also long-standing evidence that the CS glands play a broader role in regulating extracellular fluid (ECF) balance. Some of the earliest studies described how surgically removing the CS glands caused perturbations in serum levels of sodium and potassium, not just calcium [3]. Early studies also described how the glands contained a substance with vasopressor effects in both mammals and fish [4]. More recent studies have shown that hypovolemia is a potent stimulus for STC-1 secretion in fish [5] and, equally intriguingly, that the renin-angiotensin response to hypovolemia is attenuated in fish lacking STC-1 [6].

 It is also now recognized that STC-1 production is not confined to the CS glands [7–10]. Indeed the gene is variably expressed at much lower levels in most organ systems. In rainbow trout, the highest levels of expression outside the CS glands are in the male and female gonads and the anterior region of the kidney, commonly known as head kidney [4]. In flounder, *Platichthys flesus*, however, the caudal neurosecretory system has the highest levels of gene expression outside the CS glands [7]. Interestingly, the gene product in these other tissues is not necessarily the same as that in the CS glands. For instance a more heavily glycosylated variant is produced by the ovaries in female rainbow trout [11]. The function of this oocyte-derived STC-1 has not yet been addressed, but it may act locally in oocyte development and is possibly released into the circulation.

As in the case of mammals, a second form of STC, STC-2, has also been identified in fish. In the Japanese flounder, STC-2 has the same broad tissue distribution as STC-1 [12]. The functions of fish STC-2 have yet to be identified, but the limited data that is currently available suggests that, unlike STC-1, it does not modulate the movement of calcium across the fish gill [2].

The studies to date on mammalian STC-1 receptors have led to important advances in our understanding of both ligand targeting and function [13–16]. One of the most intriguing discoveries relates to manner in which STC-1 is sequestered by target cell organelles in what appears to be a receptor-mediated process. Depending on the tissue type, these organelles so far include the lipid storage droplets of adipocytes and steroidogenic cells [15, 16], the mitochondria of hepatocytes, skeletal myocytes, and nephron epithelial cells [14], and the nuclei of milk-producing mammary gland alveolar cells [13, 17]. Very little is known at present about the signal transduction and targeting pathways employed by STC-1 in fishes or if ligand sequestration takes place in this vertebrate class as it does in mammals. Therefore, the purpose of this study was to characterize STC-1 receptors in fish tissues in terms of *K*_D_ and *B*_max⁡_. We also sought to determine if fish receptors were found on mitochondria as they are in mammals. Finally, we sought to localize the receptors at the histological level, using freshwater rainbow trout as a model system.

## 2. Material and Methods

### 2.1. Animals and Tissue Preparation

Fingerling (0.5–1.0 g) and adult (200–300 g) rainbow trout were obtained from the Rainbow Springs Trout Farm in Thamesford, ON, Canda. Animals were euthanized in benzocaine dissolved in tank water for harvesting and fixation of tissues. Whole fingerling trout and tissues from adult trout were immersed in 4% paraformaldehyde and buffered to pH 7.4 with 50 mM sodium phosphate. The abdominal cavities of fingerlings were opened with a scalpel blade prior to the fixation process. Seawater-adapted Atlantic salmon kidneys bearing CS glands were obtained on arrival at the Brown's Bay Fish Packing Plant (Campbell River, BC, Canada) and placed immediately in ice-cold fixative. All tissues were then dehydrated and embedded in paraffin. Five-micron tissue sections were cut and mounted on microscope slides in preparation for localizing STC-1 protein and receptor by immunocytochemistry and *in situ* ligand binding, respectively.

### 2.2. Receptor Binding Studies

Because recombinant human STC-1 (hSTC-1) is biologically active in rainbow trout [18], receptor binding studies were carried out using a fusion protein of human STC-1 and heat-stable human placental alkaline phosphatase (STC-AP). The use of a fusion protein is necessitated by the poor binding characteristics of radioiodinated STC-1 [14]. The fusion protein has been characterized for specificity in fixed and embedded mammalian tissues by *in situ* ligand binding and in saturation binding assays on membranes, cell nuclei [13], cholesterol lipid storage droplets [15, 16], and both mitoplasts and mitochondria [14].

**Saturation Binding Assays**

Estimates of receptor *K*_D_ and *B*_max⁡_ were obtained in liver, kidney, gill, and white epaxial muscle. Each tissue was dissected from three adult trout and pooled. In the case of the gills, filaments were gently scraped with a scalpel blade to separate the soft tissue comprising the respiratory and transporting epithelia from the cartilaginous filaments. All tissues were fractionated by Dounce's homogenization and differential centrifugation for the isolation of microsomal membranes and mitochondria as previously described [14]. The relative purity of each subcellular fraction was determined using 5′-nucleotidase and succinate dehydrogenase assays as representative markers of plasma membrane and mitochondria as previously described [11].

Saturation binding assays were carried out on microsomal membranes and mitochondria from gill, liver, and muscle for estimations of *K*_D_ and *B*_max⁡_ [14]. To set up an assay, aliquots of 50–100 micrograms of membrane or mitochondrial protein were added to 1.5 mL Eppendorf tubes followed by increasing amounts of STC-AP ± 1 *μ*M recombinant hSTC-1 (all determinations were done in triplicate). Following a 90 min incubation at room temperature under constant agitation, tubes were centrifuged to pellet the membranes or mitochondria. The pellets were washed and then heat treated at 65° for 30 min to destroy endogenous AP activity. The separation of bound and free ligand, subsequent detection of AP activity, and calculations of specific binding were performed as already described [13, 14, 19]. Saturation binding curves were analyzed in GraphPad Prism (GraphPad Software Inc., San Diego, CA, USA) using a nonlinear regression analysis of one site binding to obtain best estimates of *K*_D_ and *B*_max⁡_. GraphPad was also used to obtain negative reciprocal Scatchard plots.

**In Situ Ligand Binding Studies**

To localize STC-1 receptors at the histological level, *in situ* ligand binding (ISLB) was performed as previously described [13–15, 19]. Hydrated tissue sections were incubated for 90 minutes in a 10 nM solution of STC-AP. Control tissue sections were incubated in equivalent amounts of recombinant human alkaline phosphatase (AP), also generated in MDCK cells or STC-AP plus 1 *μ*M human STC-1 or 1 *μ*M salmon STC-1. In some cases, adjacent tissue sections were stained with hematoxylin and eosin. Six fingerling trout and 3 Atlantic salmon were analyzed in this manner, and all tissue sections were evaluated by 3 different examiners.

### 2.3. Immunocytochemistry

Immunocytochemistry (ICC) was performed in select cases to determine if the distribution of STC-1 ligand corresponded with that of the receptor. ICC was done as previously described using a salmon polyclonal STC antiserum already characterized for specificity [8, 20–22]. Tissue sections were incubated overnight at 4°C with a 1 : 1000 dilution of rabbit antiserum. The sites of antibody binding were visualized with biotinylated secondary antibodies and the Vectastain ABC peroxidase detection system (Vector Laboratories, Burlingame, CA, USA). As staining controls, tissue sections were incubated in antiserum preabsorbed with salmon STC-1 (1 *μ*g/mL). All ISLB and ICC images were captured on a Nikon E1000 upright brightfield microscope (Nikon Canada, Mississauga, ON, Canada) with a DXM1200 digital camera and ACT-1 software (Nikon Canada, Mississauga, ON, Canada).

## 3. Results

**Figure 1:**
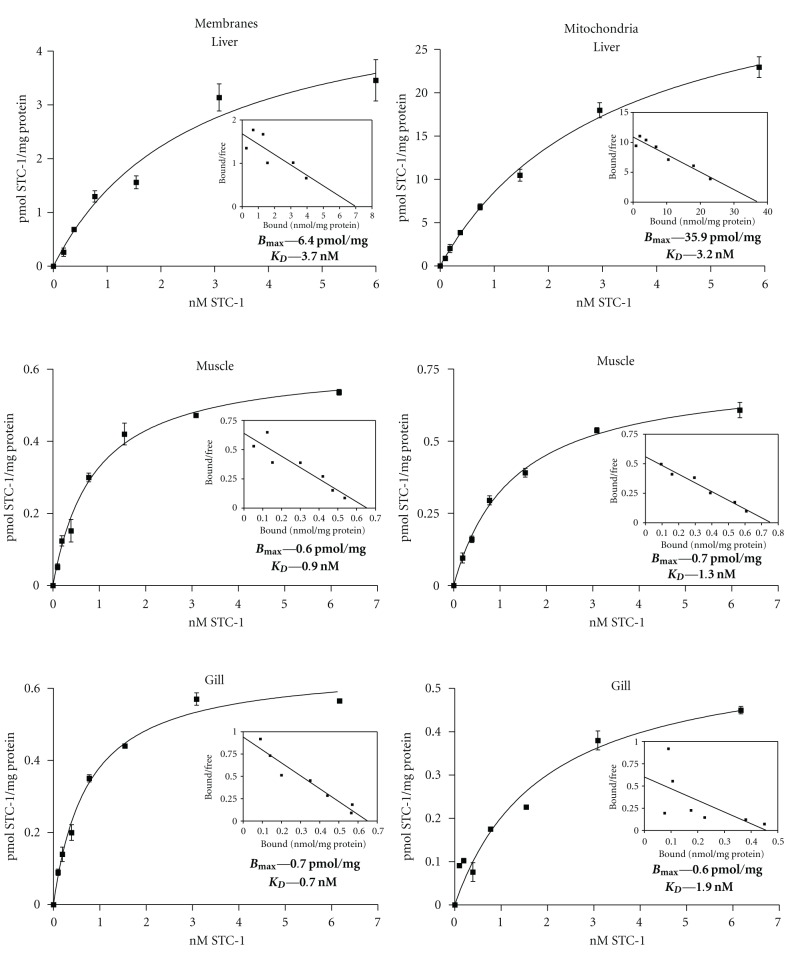
Receptor binding assays on muscle, liver, and gill. Saturation binding assays revealed the presence of saturable, high-affinity binding sites on microsomal membrane and mitochondria fractions from liver, muscle, and gill tissue from adult trout. Liver membranes and mitochondria had the highest binding capacities and lowest affinities, whereas the data for muscle and gill were similar. Each data point shown represents the mean ± SEM of three replicates. Each binding assay was repeated three times. The *B*_max⁡_ and *K*_D_ values listed in each panel are those estimated from the saturation binding curves. Scatchard plots derived from the saturation binding data are shown as insets in each panel.

Saturation binding studies were carried out on isolated microsomal membrane and mitochondrial preparations from three different tissues; liver, epaxial muscle, and the soft tissue component of the gill filament. In each instance, high-affinity and saturable binding sites were identified in both subcellular fractions (Figure 1). In mitochondria, binding capacity was highest in liver (36 pmol/mg protein) and equally low in muscle (0.7 pmol) and gill (0.6 pmol). Similarly, microsomal membrane binding capacity was highest in liver (6.4 pmol/mg) and 10-fold lower in muscle and gill (Figure 1). The binding affinities of liver microsomal membranes and mitochondria (3.2–3.7 nM) were lower than those on muscle and gill (0.7–1.9 nM) but would in all instances be classified as high-affinity binding sites, as befitting the concentrations of circulation STC-1 (0.1–5.0 nM) in fish serum (33–35).

*In situ* ligand binding (ISLB) studies revealed the presence of specific, STC-1 binding sites in most tissues and/or organs. These included brain, cartilage, adipocytes, skeletal, cardiac and visceral smooth muscle, liver, kidney, gastrointestinal tract, the eye, and the teeth.

**Figure 2:**
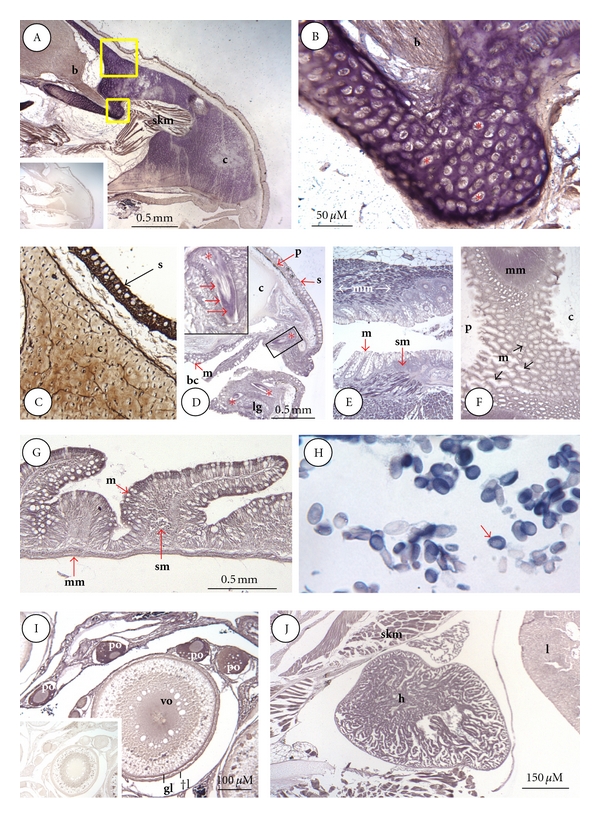
ISLB staining of snout, alimentary canal, erythrocytes, ovary, and heart. (A), Snout; STC-1 binding sites in the snout are most prominent in cranial cartilage (c), the inset at the lower left is a staining control; (b), brain olfactory bulb; skm, skeletal muscle. (B), higher magnification of smaller boxed area in (A) showing intense binding activity confined to the matrix surrounding lacuna-bound chondrocytes (*). (C), tissue section adjacent to larger boxed area in (A), stained by ICC for STC-1 protein. In cranial cartilage (c), STC-1 is present in both chondrocytes and the surrounding matrix. High immunoreactivity is also present in skin (s). (D), Buccal cavity; binding sites are most evident in the mucosal (m) layer of cells lining the mouth and emerging teeth (*), the boxed area shows a magnified tooth (*) with high binding activity (red arrows) in the dentine layer (bc, buccal cavity; s, skin; p, pigment layer). (E), esophagus; weak binding is present in the mucosal (m) and sub-mucosal (sm) layers of the esophagus. Muscularis mucosa (mm) exhibits the highest binding activity. (F), stomach; binding is lowest in mucosa (m) and highest in the underlying muscularis mucosa (mm) at the junction of the cardiac (c) and pyloric (c) regions of the stomach. (G), small intestine; binding is highest in mucosal enterocytes (m) and muscularis mucosa (mm) and lowest in the submucosa (sm). (H), red blood cells; most erythrocytes contain high binding activity that is confined to the cytoplasm. (I), ovary, the highest binding is confined to the cytoplasm of primary oocytes (po). Weaker binding is evident in theca cell (tl), granulosa cell layers (gl), and cytoplasm of vitellogenic oocytes (vo). The inset at the lower left is a staining control. (J), heart (h); all cardiac myocytes exhibited high binding activity as did surrounding skeletal myocytes (skm). Lower binding was present in liver (l) hepatocytes.

Figure 2A shows the distribution of binding sites in the snout of a fingerling trout. The different shades of staining obtained by ISLB are indicative of binding density; brown representing the lowest number of binding sites, purple being intermediate, and blue-black being the highest. Of all the tissue types comprising the snout, the underlying cartilage (C) of the developing cranium exhibited the most intense binding activity. In the specimen shown, binding activity was most intense in the regions bordering the brain (Figure 2A). At higher magnification, however (Figure 2B), it was evident that the majority of STC-1 binding sites were not in chondrocytes, but rather the surrounding matrix (Figure 2B). The degree of cranial binding activity varied greatly throughout the cranium and among individual fish (compare Figures 2A and 2C). The same was true in the case of the appendicular skeleton (not shown). Skeletal muscle fibres within the snout also exhibited strong binding activity, as did nerve fibres within the forebrain (Figure 2A). The binding of STC-AP was abolished with the inclusion of recombinant hSTC-1 in the incubation (Figure 2A inset).

Figure 2C through 2G in Figure 2 show the distribution of STC-1 binding sites in the alimentary canal, beginning with the buccal cavity. In the buccal cavity (Figure 2C), the epithelial lining and underlying muscularis mucosa exhibited moderate binding activity. Of particular note were the developing teeth where strong binding activity was apparent in the dentine layer. In contrast, the underlying pulp exhibited little or no binding activity (inset in Figure 2C). The mucosal epithelium lining the buccal cavity was primarily made up of goblet cells, all of which exhibited moderate binding in their basolateral surfaces (Figure 2D), whereas the underlying submucosa exhibited weak binding. By far the highest binding was observed over visceral smooth muscle cells comprising the underlying muscularis mucosa (Figure 2D). The same pattern of receptor binding activity was apparent in the esophagus (Figure 2E) and particularly in the stomach (Figure 2F), where the more highly developed muscularis mucosa exhibited the strongest binding activity. Only in the small intestine did mucosal epithelial cells begin to show significant binding activity (Figure 2G). Overall, therefore, binding activity in the alimentary canal was highest in visceral smooth muscle cells (muscularis mucosa) in all segments anterior to and including the stomach.

Red blood cells exhibited some of the most intense binding activity (Figure 2H). In all cases the cell nucleus was negative as compared to the cytoplasm where binding was evenly distributed throughout. In all specimens and age classes, the majority of erythrocytes (>80%) were stained in this manner.

Within the ovary, the cytoplasm of previtellogenic oocytes in particular displayed high binding activity (Figure 2I). Oocyte nuclei exhibited little binding activity by comparison. With each stage of maturation, the degree of binding within the cytoplasm decreased markedly, such that in vitellogenic stage the majority of receptors were confined to the center of the oocyte. Binding was also evident in the surrounding theca and granulosa cell layers of vitellogenic oocytes. STC-AP binding to these ovarian cell types was reduced markedly by the inclusion of recombinant hSTC-1 (Figure 2I inset).

As in the case of visceral smooth muscle, there was strong binding to cardiac myocytes, but less so to liver hepatocytes (Figure 2J).

**Figure 3:**
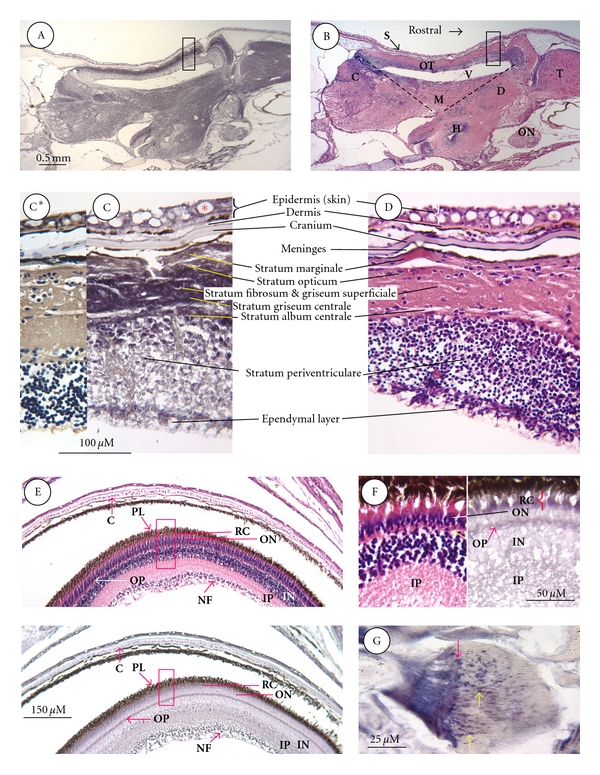
ISLB staining of brain, eye and scale. (A)–(D) Brain; adjacent sagittal sections through the trout fry head stained by ISLB for receptors (A) or with hematoxylin & eosin (B). Binding is highest in regions of the brain comprising neuropil areas and nerve tracts (pink) and lowest in areas comprising neuronal cell bodies (blue). The boxed areas in (A) and (B) are magnified in (C) and (D), respectively. Highest binding activity is evident over neuropil and fibre areas in the outer aspect of optic tectum (OT), comprising strata marginale, opticum, fibrosum, griseum, and album centrale. In contrast, relatively weak binding was present over cell bodies in the ventral aspect of optic tectum comprising stratum periventriculare and the ependymal layer. (C*) is a tissue section adjacent to (C), stained by ICC for STC-1 protein. Most of the STC-1 immunoreactivity is over neuropil and nerve tracts in the inner aspect of optic tectum. (E) and (F), Eye; adjacent sections through the trout fry eye stained with hematoxylin & eosin (top in E) or by ISLB for receptors (bottom in E). The boxed areas in (E) are magnified in (F). Binding is highest in the neuropil layers—inner plexiform (IP) and outer plexiform (OP), nerve fiber (NF), rods and cones (RC)—and weakest in layers comprising small cell bodies—inner nuclear (IN) and outer nuclear (ON). Other abbreviations; C; chondrocyte; NF, nerve fibre layer; PL, pigment layer. (G). Scale; strong ISLB staining is evident over radii in the anterior (yellow arrows; right) and posterior fields (red arrow; left).

Figure 3 shows the distribution of STC binding sites in a representative region of the brain and a cross-section through the eye of a fingerling trout. Figures 3A and 3B show adjacent sagittal sections through the right side of the brain encompassing the optic tectum overlying the mesencephalic ventricle and stained for receptors (A) and with haematoxylin-eosin (B), respectively. The general trend revealed by comparing the two sections is that receptor binding activity is highest in regions composed primarily of neuropil (pink in (B)) and lowest in regions rich in cell perikarya (dark blue in (B)). Optic tectum, which had the highest binding activity, is a case in point. The layers made up almost entirely of neuropil and fibres exhibited the highest binding activity (between stratum marginale and stratum album centrale in Figures A and C). In comparison, the inner layer of optic tectum comprised entirely of cell bodies and their nuclei exhibited little or no binding activity (stratum periventriculare in A and C). Exactly the same trend was evident outside the optic tectum, where strong binding activity was observed over neuropil-rich regions and nerve tracts comprising the granular eminence of the cerebellum (C), midbrain (M), diencephalon (D), telencephalon (T), and the optic nerve (ON), while weaker binding was seen over zones rich in cell bodies. For this reason, the inferior lobe of the hypothalamus (H) stood out on account of having weak binding activity, but this was only due to the high concentration of cell nuclei in this particular tissue section.

Figures C and D in Figure 3 are higher magnifications of the optic tectum, stained for receptors and haematoxylin eosin, respectively. Within the optic tectum, neuropil and fibre regions comprising the strata marginale and opticum, strata fibrosum and griseum superficiale, as well as the strata griseum central, and album central, exhibited intense binding activity. It was difficult to determine if binding sites were similarly present on the neuronal cell bodies (fewer in number) in these same strata. Strong binding activity was evident, however, on cell bodies in the outermost aspect of the stratum periventriculare and in the ependymal layer. These receptor-positive cells in both zones also had in common a strong degree of eosinophilia, indicative of neuropil and fibre regions.

The skin overlying the cranium exhibited moderate levels of binding activity, specifically the cytoplasm of mucous-secreting cells, whereas little or no binding was evident in the dermis or the cartilaginous cranium (Figure 3C).

Figures 3E and 3F are a cross-section through the fingerling eye, stained with either STC-AP or haematoxylin-eosin and photographed at low (E) and high magnification (F). There was no receptor binding activity of note in the lens or cornea. Binding activity was confined for the most part to rods and cones (RC), the outer plexiform (OP), inner plexiform (IP), and nerve fiber (NF) layers. Some inner nuclear (IN) layer cells also showed clear binding activity. The cytoplasm of all chondrocytes (C) within the cartilaginous optic capsule was receptor positive, as was that of cells comprising the outer epithelial capsule. Finally, Figure 3G shows a single ctenoid-type scale from a fingerling trout where strong binding activity is evident in the radii.

**Figure 4:**
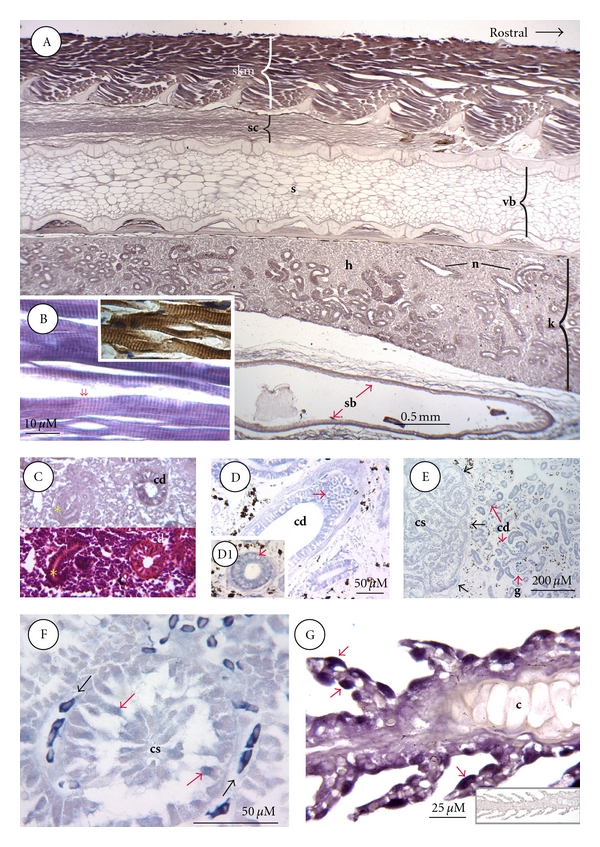
ISLB staining of trunk, muscle, kidney, corpuscle of Stannius, and gill. (A) Dorsoanterior trunk; this sagittal section shows that the highest ISLB staining is over epaxial skeletal muscle (skm), followed by spinal cord (sc), nephron (n), and the outer muscularis layer in swim bladder (sb). Hematopoietic tissue (h) within the kidney (k) exhibits weak binding activity. Other abbreviations; s, stromal adipocytes; vb, vertebral body. (B) Skeletal muscle; at high magnification it is evident that STC-1 binding is present over regularly spaced striations that are reminiscent of Z lines (1.5 *μ*M between the red arrows). The inset in panel (B) is an adjacent section stained by ICC, showing STC-1 protein sited over the same regularly spaced striations. (C) Developing kidney tubule; adjacent sections stained by ISLB (top) or hematoxylin & eosin (bottom) reveal a collecting duct (cd) and a developing kidney tubule (yellow asterisk). ISLB staining is evident over collecting ducts but not developing tubules. (D) Atlantic salmon collecting duct; STC-1 binding sites are evident in both the cytoplasm and nuclei (red arrow in D) of collecting duct cells (cd), in contrast to distal tubule cells where binding is cytoplasmic and not nuclear (red arrow in D1). (E) Atlantic salmon corpuscle of Stannius (CS; left) and adjacent kidney tissue (right); binding is strongest to kidney tubules and weaker over STC-1 cells of CS gland (defined by black arrows). Abbreviations: cd; collecting duct; g, glomerulus. (F), Atlantic salmon corpuscle of Stannius (CS) in panel (E) at higher magnification; ISLB staining is highest in red blood cells (black arrows). Staining is also evident over individual cells comprising the CS lobule (red arrows). (G) Gill filament; binding activity is highest in the cytoplasm of isolated cells on and between the primary lamella (red arrows). Staining was absent over filament cartilage (c) and weak elsewhere. The inset at lower right is a staining control.

Figure 4A is a sagittal section through the anterior trunk of a fingerling and stained for receptors. As in the case of skeletal muscle elsewhere, binding activity was highest in the epaxial musculature. At higher magnification it was evident that this binding was confined to regularly spaced striations that spanned the sarcomere at intervals of ~1.5 microns (Figure 4B). Correlative ICC staining revealed that STC-1 protein was similarly sited over the striations (Figure 4B; inset). The cartilaginous neural spines of the vertebral column that protruded into the epaxial musculature were comparatively devoid of binding activity, as was the cartilage comprising each vertebral body (Figure 4A). However, weak binding was present in the underlying stromal adipocytes and skeletogenic layer of cells coating each vertebral body. In addition, like the nerve tracts throughout the brain, those in the spinal cord contained binding activity (Figure 4A).

Nephron epithelial cells were generally receptor-positive in both the anterior and posterior portions of the kidney. The one notable exception was newly developing nephron segments, which were receptor-negative (Figure 4C). For comparative purposes, kidney tissue was also examined from a seawater-adapted salmonid, the Atlantic salmon. Developing nephron segments were not found in adult salmon, and all tubule types proved to be receptor-positive. Furthermore, as in freshwater trout, hematopoietic tissue was generally devoid of binding activity. Intriguingly, however, whereas receptor binding was confined to the cytoplasm of most nephron cell types, both nuclear and cytoplasm binding activity were clearly evident in salmon collecting duct cells (Figure 4D).

Unexpectedly, there was low, albeit significant, binding activity in STC-1 producing cells from the corpuscle of Stannius of both salmon and trout. Whereas all CS cells exhibited a low level of binding activity (Figure 4E), a sub-set of cells in most lobules displayed a visibly higher level of binding (Figure 4F).

Finally, an examination of gill filament revealed the presence of intense binding activity on a group of cells that were highly reminiscent of chloride cells in terms of their larger size and location on secondary lamellae. Ligand binding to these cells was exclusively over the cytoplasm (Figure 4G).

## 4. Discussion

Previous studies have demonstrated that the STC-1 gene is widely expressed in fish, in tissues other than the CS glands [7–10]. The present report has now revealed that the receptor is as widely distributed as the ligand and that they likely mediate paracrine and autocrine signalling in a wide range of tissues. However, which tissues are targeted exclusively by blood-borne versus locally produced ligand still needs to be established.

STC-1 receptors have, thus far, been described in mammals, and leeches [2]. In both cases the receptors and/or binding sites were saturable and had reported affinities in the low nM range depending on tissue and organelle type. Leech receptors have a somewhat lower affinity with a *K*_D_ in the 10 nM range [2]. The fish receptor likewise proved to be saturable, had affinities that fell well within the range of those reported in mammals and was found to be present on both microsomal membranes and mitochondria. The latter finding in particular is compelling evidence that the fish ligand targets the mitochondrial compartment, at the very least in hepatocytes, myocytes, and gill filament cells. However, the number of cell types targeted in this manner is likely to be considerably larger.

As in the case of many polypeptide hormones, STC-1 has different effects on different cell types [2, 17, 23, 24]. Thus, it is not altogether surprising that it is targeted to a wide range of cell and tissue types. Moreover, where a receptor is located can often be indicative as to function. In fish cartilage, for instance, the STC-1 gene is expressed in chondrocytes [8]. However, in the present study, the receptor was preferentially sited in the cartilage matrix, with much lower levels being found in nearby chondrocytes. Correlative immunocytochemical staining showed that the ligand was present in both chondrocytes (the site of synthesis) and matrix. Thus, it would appear that STC-1 receptors are tethering STC-1 ligand within the cartilage matrix, a scenario that is highly reminiscent of TBG-*β* and the means of its storage in mammalian bone. TBG-*β* is deposited within the bone matrix tethered to latent TGF-*β* binding protein. When the ligand is subsequently liberated during osteoclastic bone resorption, it then attenuates further osteoclast activity [25]. As in all vertebrates, matrix remodelling is a requisite feature of fish growth and development. Hence, it is possible that the STC-1 sequestered within the matrix has a regulatory role in the remodelling process, perhaps by acting back on chondrocytes in an autocrine feedback loop. This certainly appears to be the case in mammals. The mammalian STC-1 gene is similarly expressed in chondrocytes. And yet in cultured rat metatarsals, additions of STC-1 attenuate growth plate development by slowing chondrocyte growth and matrix synthesis [24]. Similarly, STC-1 overexpression in transgenic mice delays skeletal development and cranial suture closure [23]. Hence, there is already good evidence in mammals of roles for STC-1 in cartilage and cranial development. The absence of a bone phenotype in STC-1 null mice, which was somewhat unexpected, is likely indicative of redundancies in these critical developmental pathways [26]. And, from a comparative perspective, there may be functional significance to the fact that STC-1 and its receptor in mammalian cartilage are preferentially sited in chondrocytes [24], as opposed to the surrounding matrix as in fishes.

In the case of neural tissues, such as the brain, spinal cord, and eye, the majority of STC-1 receptors were associated with nerve tracts rather than cell bodies. Most notable of the brain regions examined was the heavy staining over neuropil and fibre areas comprising the optic tectum, the main visual center. The selective targeting of STC-1 to these areas could be reflective of any number of roles. For instance, STC-1 has proven to be cytoprotective in mammalian neurons exposed to hypoxia, in part by increasing cellular phosphate uptake and reducing cytosolic calcium levels [27]. As such, the hormone could easily be acting as an antiapoptotic agent. Alternatively, through its effects on mitochondrial calcium uniport activity STC-1 could be regulating cytosolic calcium levels as a means of controlling neuronal excitability. More detailed studies on the fish brain are clearly warranted by the present findings. They are justified more so by a second major difference in mammalian and piscine receptors, namely, in their distribution patterns on neuronal cells. In the regions of mammalian brain analyzed to date, the large majority of STC-1 receptors are on the neuronal cell bodies and not their nerve tracts [28]. It is possible, however, that the receptor distribution in the brains of trout fry will become more “mammalian-like” upon adulthood and that the dissimilarities between mammals and fish are simply due to developmental stage.

Many of the tissues containing STC-1 receptors were transporting epithelia as befitting the role of the hormone in ion transport. Receptors were present along the entire length of the alimentary canal, in mucosal epithelial cells lining the buccal cavity and esophagus, and were particularly abundant in enterocytes where STC-1 promotes luminal HCO3^−^ secretion [29]. Coincidently or not, high receptor levels were also associated with the red blood cell fraction that plays a significant role in acid-base balance via the Cl^−^/HCO3^−^ exchanger [30]. In the gills, another significant site of HCO3^−^ exchange, STC-1 receptors were most evident on cells that had the hallmarks of mitochondria-rich, chloride cells (larger cells, individually sited, on or between secondary lamellae). The majority of these receptors were cytoplasmic and distinctly nonnuclear. Calcium uptake by the gills occurs principally via epithelial channels (ECaC) on chloride cells and pavement cells [31], and the most recent evidence shows that STC-1 negatively regulates ECaC gene expression as a means of attenuating gill calcium transport [10]. Whether or not regulated calcium uptake occurs via both cell types remains to be seen, but the present data would indicate that STC-1 preferentially targets chloride cells. Gill mitochondria also contained saturable, high-affinity STC-1 receptors. Most of these were undoubtedly derived from chloride cells, which have the greatest number of mitochondria on the respiratory epithelium as befitting their central role in ion transport. If STC-1 ultimately proves to regulate chloride cell ECaC activity (as opposed to only ECaC gene expression), the mechanism could very well involve the mitochondria. In mammalian mitochondria, STC-1 increases the mitochondrial uptake of calcium [32]. In some cell types, the underlying purpose of the effect is thought to be cytoprotective, due in part to reductions in cytosolic calcium [27]. However, reductions in cytosolic calcium also affect cellular events such as ion transport. In mammalian kidney, the activities of both the Na^+^/H^+^ exchanger and NaCl cotransporter require high intracellular calcium levels for maximal rates of transport [22, 33]. Viewed in this light, the targeting of chloride cell mitochondria could be the means by which STC-1 regulates ECaC activity.

With respect to other tissues involved in mineral balance, *in situ* ligand binding revealed the presence of receptors in most nephron segments. In most instances they were cytosolic as is the case in mammalian kidney [14, 19]. In the collecting ducts of Atlantic salmon, however, they were more heavily concentrated along the lumen, indicative of a role in luminal transport. Another unique feature of Atlantic salmon collecting duct cells was in their nuclear binding activity. Nuclear STC-1 receptors are also found on milk-producing alveolar cells in the pregnant and lactating rodent mammary gland [13]. Here they play critical roles in mammary development and milk fat synthesis, both of which are compromised by interruptions in STC-1 targeting [17]. The prominence of nuclear receptors in the marine fish may be indicative of a role in the renal excretion of divalent cations. Developing nephron segments were notable for their lack of receptors, a finding that is particularly intriguing because they have the highest levels of STC-1 gene expression in trout kidney [8]. This would suggest that any ligand produced therein is destined for targeting elsewhere. Such a target could be adjacent, more fully differentiated nephron segments that are known to contain ligand but little or no STC-1 transcript [8] and have only now been shown to possess the receptor. This implies that STC-1 from developing nephron segments could be targeted to nearby cells in more mature tubules. This type of mesenchymal-epithelial signalling has already been described during mouse nephrogenesis, whereby STC-1 from mesenchymal cells is targeted to developing collecting duct cells [34]. The underlying purpose of the mammalian pathway is not understood, and yet it is possible that a similar scenario is operative in fish.

Intriguingly, STC-1 receptors were also prominent on the radii of scales (anterior and posterior). Presumably, they are used as a means of tethering STC-1, as scales also contain STC-1 immunoreactivity in a distribution pattern that is not unlike that of its receptor (Wagner, unpublished). Scales are an abundant source of calcium that can be mobilized by the piscine homologues of PTH and PTHrP [35, 36]. Given its role as an antihypercalcemic agent, STC-1 would likely oppose the actions of both peptides.

The presence of STC-1 receptors in corpuscles of Stannius cells can be interpreted in a number of ways. It could be the means by which secreted STC-1 feeds back on the cells of origin, similar to vasopressin feeding back on the neural lobe to regulate its own release. Alternatively, it is possible that STC-1 is normally released in a tethered state, complexed with a soluble form of receptor, or binding protein. At present, there is no evidence for STC-1 binding activity in fish blood outside the red blood cell (RBC) fraction. However, this is not the case in mammals. In addition to receptor-bearing RBCs, mammalian serum contains a soluble binding protein that is often seen in glomerular filtrates [19]. Binding proteins can serve many roles, including that of controlling the bioavailability of circulating ligands. Indeed, the existence of a serum binding protein could explain why the STC-1 in fish blood undergoes such wide ranging changes in its bioactivity [20]. As such, a binding protein, coreleased in greater or lesser amounts with STC-1, could represent an additional level of control over ligand action and/or function.

Because the STC-1 receptor has yet to be purified or cloned, its physical structure and downstream signalling pathways are essentially unknown. There is some evidence to suggest that the receptor is G-protein coupled. In flounder proximal tubules, the effects of STC-1 on apical phosphate transport are protein kinase A dependent (blocked by H-89 and mimicked by forskolin) and accompanied by a doubling in cAMP output [37]. Other than this one study, however, there is no other corroborative evidence in mammals or fish to support the notion that the receptor is indeed G-protein coupled. Another intriguing facet of the mammalian receptor relates to its role in ligand sequestration within target cell organelles. Ligand sequestration occurs in mitochondria, nuclei, and lipid storage droplets and accounts for target cells having such high levels of STC-1 immunoreactivity in the absence of any measurable gene expression [2]. In the case of mitochondria, there is an ever growing list of peptide hormones that are sequestered therein; angiotensin II, TGF-*β*, growth hormone, and atrial natriuretic peptide, to name only some [38]. Most of the studies reporting these findings have been done in mammals. However, the demonstration of STC-1 receptors on fish mitochondria now argues for a much wider utility of this pathway among the vertebrates.
